# A narrative review on the psychological mechanisms and efficacy of music interventions for improving symptoms of patients with ADHD

**DOI:** 10.3389/fpsyg.2026.1726801

**Published:** 2026-03-10

**Authors:** Yanyu Zhu, Dongmei Chen, Wu Chen

**Affiliations:** 1 School of Arts, Wuhan University, Wuhan, China; 2 Wuhan University Youth Research Center, Wuhan, China; 3 School of Marxism, Wuhan University, Wuhan, China; 4 Institute of Development and Educational Psychology, Wuhan University, Wuhan, China

**Keywords:** music interventions, ADHD, neuro-mechanism, non-pharmacological treatment, cognitive-behavioral therapy

## Abstract

**Objective:**

This study aimed to review the research progress on the use of music interventions for improving symptoms of attention deficit hyperactivity disorder (ADHD) and to clarify its potential mechanisms and clinical value.

**Method:**

Using the keywords “music interventions” and “ADHD,” we systematically surveyed empirical studies published in the past decade and summarized evidence from four domains: neuro-mechanisms, attention, impulse control, and emotion regulation.

**Results:**

Music interventions enhances sustained attention, reduces hyperactive and impulsive behaviors, and alleviates negative emotions such as anxiety and irritability by activating reward pathways, increasing inter-hemispheric brain synchrony, and modulating dopaminergic and noradrenergic function. Both active instrumental performance and passive music listening produce benefits, and combined use with cognitive-behavioral therapy can further improve efficacy.

**Conclusion:**

As a safe, low-side-effect, and highly acceptable non-pharmacological approach, music interventions can effectively relieve core ADHD symptoms. Future studies should establish standardized and individualized protocols through large-sample randomized controlled trials to facilitate clinical translation.

## 1 Introduction

Attention deficit hyperactivity disorder (ADHD) is a neurodevelopmental disorder that originates in childhood. According to the Diagnostic and Statistical Manual of Mental Disorders, Fifth Edition (DSM-5), the diagnostic criteria for ADHD include persistent symptoms of inattention, hyperactivity, or impulsivity that occur across multiple environments (e.g., home and school), significantly impacting the individual’s social, academic, or occupational functioning (American Psychiatric Association, 2013; Banaschewski et al., 2024). ADHD affects approximately 6 to 7% of children aged 7 to 12 years, and this prevalence decreases to 5.6% in adolescents aged 12 to 18 years (Salari et al., 2023; Franke et al., 2018). However, 50.9% of children diagnosed with ADHD continue to experience symptoms into adulthood, with the disorder having a significant impact across the lifespan (Hinshaw et al., 2024). ADHD often impairs academic performance and daily functioning; furthermore, it may lead to long-term social and emotional challenges, as identified in prior studies (Sihvonen et al., 2017; Banaschewski et al., 2024; Goes et al., 2025.

In recent years, music interventions have gained increasing attention as a non-pharmacological treatment for ADHD. ADHD, supported by emerging systematic reviews and clinical studies. For instance, a 2025 systematic review covering studies from 1981 to 2023 identified a substantial and growing body of work exploring music therapy and listening interventions aimed at reducing ADHD symptoms. Similarly, recent reviews have highlighted neurocognitive mechanisms through which both active and passive music interventions may improve attention, self‑regulation, and other ADHD‑related impairments (Jiang et al., 2025; Yang et al., 2025; Iordache, 2024).This therapeutic approach utilizes structured music stimuli to modulate neural pathways associated with attention, emotion regulation, and behavioral control (Xiao et al., 2023a). Luo and Zhang (2025) proposed a systematic framework for understanding seven key neuro-mechanisms by which music interventions improve ADHD symptoms, including enhancing executive function, improving time perception, regulating brain arousal states, optimizing default mode network activity, promoting neural oscillation synchronization, assisting in emotion management, and improving social connectivity (Park et al., 2023).

Music interventions is a goal-oriented and highly structured interactive process that uses musical materials to guide ADHD patients toward positive changes in cognition, emotions, and behavior (Desai et al., 2024). Intervention methods can be divided into receptive music interventions, creative music interventions, improvisation, recreational music performance, and music games, with multi-level strategies for therapy. From the perspective of participant motivation, interventions can be categorized into active and passive forms (Dan et al., 2025). Active music interventions emphasize the individual’s active participation, such as instrument playing and singing, which enhance attention control and self-regulation through real-time music creation (Martin-Moratinos et al., 2023). In contrast, passive music interventions focuses on placing ADHD patients in a music environment where they listen to specific types of music for therapeutic effects. Kausel et al. (2023) found that ADHD patients show significant improvements in reading comprehension and attention when listening to rhythmically stable and calming music, which enhances concentration and reduces impulsivity (Woods and Clark, 2024). Despite the promising potential of music interventions, current research still faces challenges such as small sample sizes, a lack of long-term follow-up data, and the absence of standardized intervention protocols. Although the above research reveals the great potential of music interventions, practical implementation and theoretical advancement in this field still face notable challenges. There is a pressing need for more scientific and systematic research to advance the clinical practice of music interventions for ADHD patients (Zemestani et al., 2023).

## 2 Methods

### 2.1 Literature search and inclusion criteria

A comprehensive literature search was conducted using database. including PubMed, Scopus, Embase, and Chinese academic databases. The keywords used for the search were “ADHD,” “music intervention,” “rhythmic training,” and “randomized controlled trial” to identify relevant studies. The studies included in the review were required to meet several inclusion criteria: they had to be published within the last 10 years, focus on ADHD patients, and evaluate the effects of music-based interventions on ADHD symptoms. Outcome measures considered for inclusion included behavioral, cognitive, or neurophysiological indicators related to attention, impulsivity, or emotional regulation. Furthermore, only peer-reviewed studies with full-text availability were included. Studies were excluded if they did not include control groups, lacked a clear comparative framework, or merely described intervention protocols without reporting specific measurable outcomes. Additionally, studies that focused on populations outside of ADHD patients were not considered for inclusion.

### 2.2 Data extraction

Data from eligible studies were systematically extracted based on several key factors that were deemed relevant to understanding the effectiveness of music interventions for ADHD. We focused on the type of intervention used in each study, including passive music listening, rhythmic training, and active music therapy. The characteristics of the population were also extracted, including age range and ADHD subtype (such as inattentive, hyperactive-impulsive, or combined). Information about the intervention parameters was recorded, such as the type of music used (e.g., classical, rhythmically stable music), the duration and frequency of the intervention, and the delivery method (whether the intervention was passive or active). Lastly, we extracted data on the outcome measures used in each study, which included attention tasks, emotional regulation scales, and neurophysiological markers such as heart rate and cortisol levels. Studies that did not provide data on any of these outcome measures or lacked clear descriptions of their intervention protocols were excluded from the data extraction process. This approach ensured that only relevant and high-quality studies were included in our review.

### 2.3 Quality assessmen

The quality of each study included in this review was assessed based on several criteria to ensure that only robust and reliable evidence was considered. We focused on factors such as sample size, study design, outcome measures, and the risk of bias. The sample size of each study was evaluated to determine whether it was large enough to provide meaningful results and avoid statistical power issues. Given that randomized controlled trials (RCTs) are considered the gold standard for clinical research, we gave priority to studies using this design. We also reviewed the outcome measures used in each study to ensure they were valid, reliable, and appropriate for measuring the key symptoms of ADHD. In terms of assessing study quality, we considered potential sources of bias, including selection bias, performance bias, and detection bias. Each study was appraised for its methodological rigor, and studies were ranked as high, medium, or low quality based on these criteria. The quality assessment was carried out manually by two independent reviewers to ensure consistency and minimize errors, though no formal software tools were used in the process.

## 3 Result

### 3.1 Music intervention principles and its alignment with ADHD pathological mechanisms

#### 3.1.1 Neuroscientific basis of music intervention

The neuroscientific basis of music intervention lies in its ability to influence brain activity and plasticity through various pathways (Thaut et al., 2015; Menon and Levitin, 2005). The rhythmic and melodic elements of music affect brain activity at multiple levels. First, music activates the auditory cortex, emotional-related areas, and motor cortex, triggering a series of neurophysiological responses (Zhu, 2022; Zhang et al., 2025a). Meanwhile, music stimulation can induce the brain to release neurotransmitters such as dopamine, which play a key role in the reward system and can enhance pleasure and motivation (Zemestani et al., 2023; Saqib et al., 2025). Music also modulates the autonomic nervous system by lowering cortisol levels and suppressing cardiovascular stress responses, thereby promoting relaxation of both the body and mind (Lou et al., 2025; Sun and Meng, 2025).

The neuroplastic changes induced by music stimulation are another important mechanism of music intervention. Studies have shown that music training and music intervention can promote the restructuring of brain structures and functions (Rickson, 2006). Music training increases the volume of brain gray matter and improves the efficiency of neural network connectivity, with this change being particularly prominent in individuals who undergo long-term music training (Hinshaw et al., 2024). Music intervention also enhances neuronal connections and synchronization, improving cognitive function and emotion regulation (Park et al., 2023). In terms of neuroplasticity, music intervention promotes the recovery of impaired neural functions by modulating neuronal excitability and synaptic plasticity (Michelini et al., 2022). In patients with brain injury, music intervention activates the brain’s default mode network, aiding in the recovery of consciousness (Xiao et al., 2023a).

#### 3.1.2 Mechanisms of music intervention in ADHD treatment

Dopamine and other neurotransmitter dysfunctions are the primary causes of ADHD. ADHD medications, such as amphetamines and methylphenidate, improve ADHD symptoms by inhibiting dopamine and norepinephrine reuptake, thus prolonging their presence in the brain. Music intervention can replace or assist medications in performing this task (Sholeh and Supena, 2021; Khorshid et al., 2024). The imbalance of neurotransmitters such as dopamine and norepinephrine results in ADHD patients’ poor performance in regulating attention, impulse control, and emotional stability (Pasqualitto et al., 2023). Dopamine dysfunction causes individuals to be insensitive to delayed or abstract rewards, making it difficult to gain satisfaction and motivation from everyday tasks (Sihvonen et al., 2017). Insufficient dopamine in the striatum also impairs behavioral inhibition (Banaschewski et al., 2024). Music activates the brain’s reward system, elevates dopamine levels in ADHD patients, and thereby improves the “braking” system, enhances behavioral inhibition, and long-term training effectively stabilizes emotions, reducing impulsive and hyperactive behaviors (Luo and Zhang, 2025; Thaut et al., 2015). Norepinephrine dysfunction leads to inefficient neural signal transmission and poor neural network synchronization. Like a malfunctioning conductor, the brain cannot effectively coordinate resources. Music’s efficient use of norepinephrine can improve neuronal response speed, ignore irrelevant distractions, and help ADHD patients maintain attention more stably (Xiao et al., 2023a). Table 1 summarizes four interrelated pathways and corresponding implementation notes.

**Table 1:** Mechanistic pathways of music-based interventions for ADHD and linked clinical implications.

Reference	Mechanism pathway	Key evidence	Clinical implications
Menon and Levitin (2005) and Thaut et al. (2015)	Reward/dopamine system	Music rewards activate the midbrain-limbic system, enhancing motivation and sustained engagement; ADHD music intervention reviews support the motivation–cognition pathway	Choose “pleasing/preferred” music tracks to improve adherence and training benefits,
Thaut et al. (2015) and Spiewak (2023)	Neural oscillation/rhythm synchronization	Rhythmic training and modulated music enhance the coupling between external rhythms and internal neural oscillations	Use stable beats/modulated designs during task phases to support sustained attention
Rickson (2006) and Sun and Meng (2025)	Emotion/autonomic nervous system	Music therapy increases serotonin (5-HT) levels and decreases emotional scale scores; music intervention in diverse clinical populations helps reduce stress and aids functional recovery	Stabilize emotions first and then move to cognitive tasks to enhance overall benefits
Sihvonen et al. (2017) and Menon and Levitin (2005)	Neuroplasticity/network reorganization	Music training promotes neuroplasticity and network reorganization (neuro-rehabilitation and review evidence)	Regular and adequate frequency and duration of training are crucial for effectiveness

#### 3.1.3 Enhancement of attention in ADHD patients through music intervention

The regulatory effect of music on the attention system is mainly achieved by activating the auditory cortex and prefrontal cortex. ADHD patients often face attention deficits, and music helps them concentrate by providing stable rhythms and melodies (Martin-Moratinos et al., 2023). Research shows that different types of music have varying effects. For example, classical music or rhythmically stable music significantly increases ADHD patients’ attention span (Zhu, 2022). Park et al. (2023) pointed out that ADHD patients’ attention problems are often related to the brain’s inefficiency in processing sensory information. Music intervention, through specific audio characteristics such as stable beats and harmonious melodies, helps focus the attention of ADHD patients (Pasqualitto et al., 2023). The beats in music can activate the basal ganglia and prefrontal cortex, regions that play a key role in attention regulation (Luo and Zhang, 2025). Music intervention also significantly increases attention span by reducing background noise interference (Xiao et al., 2023a). Clinical studies have found that certain types of music (e.g., classical music) can help the brain increase alpha waves (8–13 Hz), which are associated with a relaxed and focused state. These brainwaves are more stable and active, which can better trigger the patient’s high-efficiency focus state and reduce errors caused by inattentiveness (Zhang et al., 2025b).

#### 3.1.4 Suppression of impulsive behavior in ADHD patients through music intervention

The effect of music on impulse control is mainly achieved by regulating the brain’s emotional and behavioral responses. ADHD patients often exhibit high impulsivity, which is related to dysfunction in the prefrontal cortex. Music can help ADHD patients better control impulsive behavior by activating the emotional regulation centers of the brain, such as the amygdala (Rickson, 2006). Ghetti et al. (2024) revealed through qualitative research that music intervention provides ADHD patients with a non-pharmacological way to control impulsivity. Specific melodies and rhythm patterns in music can trigger the brain’s reward system and release dopamine and other neurotransmitters, which are crucial for impulse inhibition. Regular music training can serve as an external regulatory tool, where individuals can effectively suppress impulsive behavior by actively listening to or engaging in music activities when impulsive tendencies arise (Michelini et al., 2022). Research shows that after 8 weeks of structured music intervention, ADHD patients showed a significant 40% reduction in the frequency of impulsive behaviors in daily life, demonstrating the positive effects of music intervention on impulse control (Zemestani et al., 2023). Group music activities have socializing functions. When ADHD patients integrate into a group using music as a medium, they not only gain a sense of belonging and support in social interactions but also significantly improve their self-efficacy and promote personal growth and self-improvement. These socializing functions are further amplified by music’s unique social reinforcement mechanism, which aligns with the section’s focus on impulse control (Kim et al., 2011). The positive feedback from synchronization and cooperation, such as recognition and collective honor, acts as a form of social reinforcement that enhances ADHD patients’ motivation and confidence in impulse control. This music-based social reinforcement helps ADHD patients more effectively transfer learned behavioral strategies to daily life and interpersonal interactions, leading to lasting and widespread positive changes (Woods and Clark, 2024).

#### 3.1.5 Improvement of negative emotions in ADHD patients through music intervention

Music has long been recognized for its ability to regulate emotions and improve mental well-being. In the context of ADHD, music interventions play a crucial role in alleviating negative emotions, as discussed below. In addition to the typical cognitive and behavioral impairments, ADHD patients often experience significant emotional issues such as anxiety, irritability, and anger and are prone to psychological comorbidities such as anxiety disorders, bipolar disorder, and social phobia. These emotional problems further exacerbate attentional distraction and impulsive behaviors, creating a vicious cycle. The mechanism by which music regulates emotions is primarily based on its neuro-regulatory effect on the brain’s emotional centers. Martínez-Vérez et al. (2024) showed that music, through its core elements such as melody, harmony, and rhythm, can activate the limbic system, particularly the amygdala and hippocampus, which play key roles in emotional recognition, memory integration, and stress responses. For example, soothing music, such as slow tempo and harmonious melodies, can reduce excessive excitement in the amygdala, alleviating anxiety and fear responses. Rhythmically strong and structurally clear music helps promote the release and utilization of dopamine and other neurotransmitters, thereby improving emotional states and enhancing positive emotional experiences (Sun and Meng, 2025). Music intervention provides a safe channel for emotional expression and release, helping ADHD patients better recognize, accept, and regulate their emotions. In structured music therapy or daily music listening, patients gradually learn to combine their internal emotional states with the external form of music, thereby enhancing their cognitive regulation of emotions (Desai et al., 2024).

### 3.2 Application of music interventions in ADHD treatment

#### 3.2.1 Empirical research progress

Drawing on randomized and controlled studies, Table 2 summarizes representative auditory/music-based interventions for ADHD—including populations, intervention protocols, comparators, dosing, outcome measures, and key findings.

**Table 2:** Summary of representative auditory/music-based interventions for ADHD across study designs.

Authors (year)	Population and sample	Intervention type	Comparator/control	Duration/frequency	Primary outcomes and tools	Key findings
Park et al. (2023)	Children/Adolescents with ADHD (RCT)	Structured music therapy (group)	Standard/waitlist/other	12 weeks, 3 times/week	Emotion/stress: 5-HT, CDI, DHQ	5-HT ↑, CDI ↓, DHQ ↓; improvement in emotion and stress coping (*p* < 0.05/0.01)
Chen et al. (2022)	Children with ADHD					
	White noise listening (task phase)	Silence/other sounds	30 min per session	Verbal working memory task performance	Best performance under white noise, worst in silence (supports low-arousal individuals benefiting)	
Luo et al. (2023)	Children with ADHD and comorbid ODD					
	Yoga + music combined intervention	Yoga alone; music alone	8 weeks, 2 times/week	Attention/hyperactivity-impulsivity/ODD behavior metrics	Combined intervention effect size ≈ 0.92–0.93, significantly better than single interventions	
Jamey et al. (2025)	Children with ADHD	Rhythmic training (tapping/beat task)	Standard/waitlist/other	15 min per session, 4 weeks	Temporal processing/rhythm ability	Rhythm synchronization and temporal processing improvement, attention maintenance benefits
Woods and Clark (2024)	ADHD traits population	Rapidly amplitude-modulated music (passive listening)	Non-modulated music/control	30 min per session, 4 weeks	Attention/behavioral tasks	Improvement in attention performance, supports “external rhythm → neural oscillation” pathway

#### 3.2.2 Music intervention for improving attention in Patients with ADHD

Music intervention can enhance children’s attention in practical applications, partly through modulations of autonomic nervous system activity, such as reducing heart rate and increasing parasympathetic tone, which foster a calmer internal state conducive to sustained attention (McCrary and Altenmüller, 2021; Lee et al., 2024; Rickson, 2006; Saville et al., 2025). Music therapy reduces physiological stress by lowering systolic blood pressure (SBP), diastolic blood pressure (DBP), and heart rate (HR), thus providing conditions for sustained attention (Sun and Meng, 2025). Chen et al. (2022) focused on the effect of white noise on verbal working memory in children with ADHD. The study found that children with ADHD performed best when listening to white noise and performed worst in the silence condition. This suggests that white noise can optimize cognitive performance by regulating brain arousal levels. The stable tone and intensity of white noise serve as additional neural noise, amplifying neural signals and increasing neural activity, thereby modulating arousal levels and improving attention performance (Xiao et al., 2023b). Luo et al. (2023) explored the effects of combining yoga and music intervention on children with ADHD and comorbid oppositional defiant disorder (ODD). The study found that the combination of yoga and music intervention was most effective in reducing inattention, hyperactivity/impulsivity, and ODD behaviors. The effect sizes for the combined intervention were 0.9, 0.92, and 0.93, significantly higher than those of either yoga or music intervention alone. This indicates that the combination of exercise and music improves ADHD children’s behavior and cognitive functions through multiple pathways (Luo and Zhang, 2025). These findings collectively highlight that music/sound intervention, through its physiological regulatory effects, serves as an effective approach to enhancing children’s attention, with movement potentially acting as a complementary rather than central component.

#### 3.2.3 Music intervention for alleviating hyperactivity and impulsivity in ADHD children

On the one hand, the rhythm and melody of music can attract children’s attention, helping them gradually learn to concentrate, thus effectively alleviating hyperactivity symptoms. Kirk et al. (2024) found that ADHD children who listened to rhythmically stable music showed a significant increase in attention span and a reduction in hyperactive behaviors. On the other hand, interactive elements in music interventions, such as instrument playing or music creation, help children better express themselves and release pent-up emotions, thereby reducing impulsive behaviors. A systematic review and meta-analysis by Goes et al. (2025) found that music intervention has a trend of improving impulsive behavior in ADHD children. Although it did not reach statistical significance, the effect size was 1.18, showing a potential positive impact. Luo et al. (2023) further pointed out that music intervention combined with yoga had an effect size of over 0.92 in alleviating hyperactive and impulsive behaviors in ADHD children (Luo and Zhang, 2025). Desai et al. (2024) reported the effectiveness of music intervention in multimodal rehabilitation programs, where the combination of music intervention and other therapies significantly improved ADHD children’s behavior management skills (Goes et al., 2025).

#### 3.2.4 Music intervention for emotional regulation and social function in ADHD children

This section focuses on the neurophysiological correlates of music intervention in ADHD, particularly its impact on emotional regulation and social functioning. Park et al. (2023) showed that music therapy significantly improves depressive symptoms in ADHD children by activating serotonin (5-HT) and improving stress coping abilities. The 5-HT secretion in the music therapy group significantly increased (*p* < 0.001), and cortisol levels significantly decreased (*p* < 0.001), indicating that music therapy improves ADHD children’s emotional state through neurophysiological mechanisms. Psychological scale testing showed that music therapy significantly reduced scores on the Children’s Depression Inventory (CDI) and the Daily Hassles Questionnaire (DHQ) (*p* < 0.01 and *p* < 0.001), further confirming the positive effects of music therapy in improving the psychological state of ADHD children (Park et al., 2023). Aasim (2024) pointed out that music intervention activates brain regions related to attention, emotion regulation, and memory formation, promoting the release of neurotransmitters to improve emotional regulation. Music intervention also improves social function and emotional state through social interaction and emotional expression, such as rhythm training, improvisation, and music games (Aasim, 2024). Park et al.’s (2023) study compared pre- and post-intervention data and found that music intervention significantly improved ADHD children’s attention, reduced behavioral problems, and significantly improved social skills and emotional regulation.

#### 3.2.5 Diversified forms of music intervention

Music interventions can be divided into active and passive forms. Active music therapy and passive music listening are two main forms of music intervention, each with its own advantages in improving ADHD symptoms, and they complement each other effectively. Active music therapy emphasizes the patient’s direct participation, with common forms including instrument playing and singing (Song et al., 2025; Moumdjian et al., 2025; Dorris et al., 2024; Li et al., 2022). This intervention promotes the activation of multiple brain regions, especially the prefrontal cortex and limbic system, through real-time involvement in the music creation process, thereby enhancing neuroplasticity and improving executive function (Rickson, 2006). Playing instruments requires patients to simultaneously process rhythm, melody, and harmony, helping to improve their working memory and attention allocation (Luo and Zhang, 2025); singing, on the other hand, enhances emotional self-control through emotional expression and breathing regulation (Michelini et al., 2022). Previous studies showed that after 12 weeks of regular active music therapy, ADHD patients’ performance on attention–concentration tasks improved by 30%, and their emotional stability scores increased by 25% (Thaut et al., 2015).

Passive music listening, on the other hand, focuses on the regulatory role of music as an external stimulus. Common forms include background music and rhythm training. Spiewak (2023) found that listening to certain types of music, such as classical music or rhythmically stable tracks, helps reduce environmental interference and enhances sustained attention time. Rhythm training uses periodic auditory rhythms to guide neural oscillations and synchronize with external rhythms, thereby enhancing the brain’s ability to process temporal information, which positively impacts ADHD patients’ rhythm perception and time management skills (Xiao et al., 2023b). To guide practical selection of modalities, Table 3 maps common music-based options to settings, targets, strengths, and caveats.

**Table 3:** Practical taxonomy of music-based interventions for ADHD.

Reference	Intervention type	Typical setting	Direct target	Advantages	Considerations
Rickson (2006) and Park et al. (2023)	Active music therapy (instrumental performance/choir/improvisation)	Outpatient groups; school clubs	Executive function, attention, emotional regulation,	High participation, transferable to classroom/home	Requires structured process and professional staff
Zhu (2022) and Spiewak (2023)	Passive music listening/background music	Homework/self-study	Sustained attention, interference suppression	Easy to implement, high adherence	Music selection/volume should be individualized; avoid overstimulation
Chen et al. (2022)	White noise	Task/homework binding (headphones)	Verbal working memory, attention maintenance	Significant benefits for low-arousal individuals	Sound pressure/presentation style needs standardization
Thaut et al. (2015)	Rhythmic training	Training/rehabilitation room	Temporal processing, rhythm synchronization	Promotes neural oscillation coupling with external rhythms	Intensity and complexity should match task difficulty
Luo et al. (2023)	Combined interventions (e.g., yoga + music)	Outpatient courses/multimodal rehabilitation	Dual improvement in attention and behavior	Superior to single interventions	Requires higher resource input and interdisciplinary collaboration
Xiao et al. (2023b)	Music games or music-based cognitive exercises (Dong et al., 2025)	Classroom/home use	Cognitive flexibility, problem-solving	Engaging, fun, adaptable to various age groups	Needs appropriate game design and clear instructions

### 3.3 Comparison and synergistic application of music intervention with other treatments

#### 3.3.1 Comparison of music intervention with pharmacological treatment

Music interventions demonstrate unique advantages in many aspects. According to Achuthan and Khobragade (2025), music interventions influence patients’ with ADHD attention and emotions through specific audio features such as rhythm and melody, enhancing cognitive function. Long-term regular training can have the same effect as medication. Furthermore, compared to the side effects and withdrawal reactions after discontinuing medications, music intervention offers a safer, more tolerable improvement for ADHD patients (Achuthan and Khobragade, 2025). Pharmacological treatments are limited by time and location, and imported drugs are expensive. While medications can significantly alleviate symptoms in the short term, they are often accompanied by side effects such as obesity, insomnia, dizziness, and damage to other organs. There is also a risk of dependence, and symptoms may recur after stopping medication (Nazarova et al., 2022). In contrast, music interventions, when tailored to the patient’s condition, can be performed anytime and anywhere, offering greater accessibility. Nazarova et al. (2022) further stated that as a non-pharmacological, non-invasive method, music intervention has almost no significant physical side effects and is more acceptable and adherent, especially for long-term use in children and adolescents. The effects of music interventions are diverse; they can directly affect the brain through auditory stimulation or indirectly influence cognition and behavior through the emotional responses they evoke (Schwartzberg and Silverman, 2014; Simpson et al., 2013; Han et al., 2025; Saqib et al., 2025). Through structured musical activities, such as rhythm training, improvisation, and music relaxation, music intervention not only controls symptoms but also helps in the overall psychological development and social adaptation of ADHD patients (Desai et al., 2024; Park et al., 2023). It is worth noting that music intervention does not entirely exclude pharmacological treatment. Both can be used in combination to form a multimodal intervention plan, thus reducing medication dosage and side effects while enhancing overall recovery effectiveness (Zemestani et al., 2023).

#### 3.3.2 Synergistic application of music intervention with psychotherapy

Music intervention and psychotherapy show strong potential for synergy in ADHD clinical interventions, particularly when combined with cognitive-behavioral therapy (CBT), demonstrating significant treatment-enhancing effects (Zemestani et al., 2023). Psychotherapy focuses on improving an individual’s emotional, cognitive, and behavioral functions through professional psychological methods, while music intervention complements traditional psychotherapy through its unique emotional arousal and expression mechanisms. Previous studies showed that integrating music interventions into the CBT framework can effectively improve attention and memory performance in patients, with more lasting effects (Achuthan and Khobragade, 2025). A study by Leon et al. (2024) concluded that the combined intervention of music and CBT showed significant therapeutic effects in children and adolescents with ADHD, not only improving cognitive function but also increasing the overall acceptance and sustainability of treatment (Marchetti et al., 2018). In social skills training, music intervention provides a safe platform for social interaction due to its non-verbal and emotionally inclusive nature. Through music activities such as ensemble performance, improvisation, and group creation, patients can practice social behaviors in a structured and low-pressure environment, enhancing cooperation and empathy (Goes et al., 2025). Groß et al. (2022) further showed that music performance interventions could significantly improve ADHD patients’ impulse control levels, thereby improving their social interaction quality. CBT helps patients identify and adjust maladaptive thinking through cognitive restructuring, while music interventions enhance emotional regulation and behavioral inhibition through rhythm guidance, music relaxation, and emotional expression, providing “emotional scaffolding” and motivational support for cognitive interventions (Desai et al., 2024). Zhu (2022) demonstrated that compared to single treatments, the combined use of music intervention and CBT more comprehensively improves ADHD patients’ cognitive function and emotional regulation abilities. Many universities have already started integrating music into group psychological counseling, a format well received by participants. The combination of art and psychology, which is light-hearted, inclusive, and creative, is easier for children with high ADHD prevalence to accept, showing the integrative advantages of synergistic intervention strategies (Xiao et al., 2023b).

#### 3.3.3 Development of personalized music intervention plans

In clinical treatment, music interventions can serve as a supportive rehabilitation tool at home to better improve ADHD patients’ conditions (Forte et al., 2020; Peters, 2000; Martin-Moratinos et al., 2023; Güçhan, 2024). This requires the systematic and personalized customization of the intervention according to the specific conditions of ADHD patients to achieve true “precision medicine.” First, an adaptability screening for music intervention should be conducted. Not all ADHD patients respond to music interventions equally, so preliminary evaluations should include screenings of auditory sensitivity, music perception abilities, music preferences, and emotional responses to determine whether they are suitable for music intervention (Rickson, 2006). After screening, personalized music intervention plans should be developed based on the clinical subtypes of ADHD, such as inattentive type, hyperactive–impulsive type, or combined type (Desai et al., 2024), and core symptom characteristics. For patients primarily with attention deficits, the intervention should focus on rhythmically stable and structurally clear music, using rhythmic training to enhance sustained attention (Luo and Zhang, 2025). For individuals primarily with hyperactivity and impulsivity, soothing, slow-tempo music combined with breathing guidance should be introduced to assist with self-regulation and impulse inhibition (Park et al., 2023). For patients with certain musical abilities, active interventions such as instrument playing or song creation can be incorporated to enhance their achievement motivation and self-efficacy (Achuthan and Khobragade, 2025). Ultimately, based on multi-dimensional assessments and matching, a personalized “one-person, one-plan” music intervention program should be developed, enabling more precise and efficient medical support. This approach reflects the modern rehabilitation concept of transitioning from traditional generalized interventions to individualized, biopsychosocial, integrated models (Robb et al., 2025; Leon et al., 2024; Pakdeesatitwara et al., 2025; Ro et al., 2025).

Additionally, ADHD patients often perform poorly academically, with personal achievement and future development limited. In personalized music interventions, some ADHD patients exhibit strong music perception, rhythmic response, or improvisational abilities. The discovery and cultivation of such “musical talent” can not only enhance participation motivation in interventions but may also provide direction for future career development or artistic specialties, thus enhancing self-efficacy and life meaning and fostering positive identity development (Michelini et al., 2022). This music advantage-oriented approach helps move away from the traditional deficit-compensation intervention paradigm, shifting toward the stimulation of potential and long-term career support (Zhu, 2022). To facilitate implementation across clinical and educational settings, Table 4 outlines how music-based methods can be integrated with standard ADHD care pathways.

**Table 4:** Integration of music-based approaches with established ADHD interventions.

Reference	Integration object	Purpose	Implementation approach	Basis and directional conclusions
Rickson, (2006) and Menon and Levitin (2005)	CBT	Enhance engagement and strategy maintenance	Embed short-term music/rhythm modules before and after CBT (emotion-motivation scaffolding)	Music therapy improves emotional regulation and stress coping; reviews suggest synergy with psychological interventions
Achuthan and Khobragade (2025) and Menon and Levitin (2005)	Medication	Steady-state control + non-pharmacological gains	Prescriptive music/rhythm exercises alongside medication stabilization	ADHD medication as the cornerstone of treatment; music intervention as a complementary approach to enhance functionality
Chen et al. (2022) and Lou et al. (2025)	Home/school	Real-world transfer	Fixed study periods: white noise/background music via headphones; parent/teacher recordings	White noise enhances working memory during task-learning phases; high acceptability for home–school cooperation
Luo et al. (2023) and Xiao et al. (2023a)	Multimodal rehabilitation (e.g., yoga + music)	Multidimensional improvement in attention, behavior, and emotion	Outpatient courses with combined intervention; customized by patient population	Combined interventions outperform single interventions (larger effect sizes); systematic evidence supports the effectiveness of music interventions
Spiewak (2023)	Mindfulness-based therapies	Enhance focus, emotional control, and relaxation	Integrating mindfulness practices with music-based interventions	Mindfulness and music interventions show complementary effects on emotional regulation and attention
Kim et al. (2025)	Technology-assisted interventions	Improve adherence, monitoring, and personalization	Use of apps or devices for music therapy that tracks progress and adjusts intervention based on real-time data	Tech-assisted music therapy increases patient engagement and customization potential

## 4 Current research limitations and future directions

### 4.1 Limitations of current research

This study, while contributing valuable insights, is constrained by several key limitations that warrant discussion. Small sample sizes and significant differences In study designs. Most existing studies are explorator, have small sample size, and lack large-scale, multi-center randomized controlled trials (RCTs), which limits the generalizability of the findings. There are significant differences in intervention methods, duration, and assessment tools across studies, making effective comparison and integration difficult. For example, Saville et al. (2025) conducted a study with a sample size of 1,170, with 830 diagnosed with ADHD or ADD, but the sample distribution was uneven and lacked a multi-center design, limiting the generalizability of the conclusions. Park et al. (2023) conducted a study with a sample size of 36, split into an ADHD control group and a music therapy group, with 18 participants in each group. The sample size was small, and there was no long-term follow-up.

Lack of long-term tracking and mechanistic studies: Most studies focus on evaluating the short-term effects of interventions, with a lack of long-term tracking studies. Additionally, research on the specific mechanisms of music interventions, especially their neurobiological mechanisms, is still insufficient. There is a lack of systematic neuroimaging studies to validate their long-term effects. Park et al.’s (2023) study lasted only 3 months and did not track long-term effects. Matziorinis and Koelsch (2022) pointed out that the neurobiological mechanisms of music interventions are still unclear and require further investigation.

Lack of standardized music intervention protocols: On the one hand, scientific and unified operational standards for music interventions have not been established, and key elements of intervention protocols, such as music type selection, intervention frequency, and session duration, are still in the exploratory phase. In Saville et al.’s (2025) study, the types and durations of music interventions varied significantly across different experiments, which made it difficult to compare and integrate the results effectively, thus affecting the reliability and reproducibility of the conclusions. On the other hand, the lack of standardized measures also limits the promotion and application of music interventions in clinical settings, resulting in relatively low adoption in domestic practice and requiring greater social recognition and acceptance (Zhang, 2024; Sun, 2022; Tarfi, 2025).

Limitations in the use of music materials: Most existing studies use music as a whole as an intervention material, classified by music genre. Matziorinis and Koelsch (2022) pointed out that the mechanisms by which musical elements, such as consonance, tempo, and tonality, influence cognition and emotions are not well understood, and the specific mechanisms of each musical element are unclear. While comprehensive music materials are more accessible and operationally strong, it is difficult to investigate how individual musical elements affect ADHD and what their respective weightings are, limiting the precision and individualization of the intervention methods (Zemestani et al., 2023) These limitations provide directions for future research, such as addressing sample size constraints and expanding measurement methodologies to strengthen the generalizability of findings.

### 4.2 Future research directions

Promote large-sample, multi-center randomized controlled trials and further research: Future research should conduct large-scale, multi-center RCTs to enhance the credibility and generalizability of music intervention studies. Most current studies have limited sample sizes and are concentrated in single institutions. Future studies should build upon effective previous models and expand sample sizes to increase the reliability of the conclusions. Additionally, mechanistic studies should be further deepened, particularly focusing on the neurobiological pathways of music interventions. As suggested by Matziorinis and Koelsch (2022), fMRI, EEG, and other neuroimaging technologies should be widely used to systematically examine the effects of music interventions on brain structures, functional connectivity, and neural oscillation patterns in ADHD patients, providing strong scientific evidence for clinical applications of music therapy (Matziorinis and Koelsch, 2022).

Strengthen the integration of music interventions with other treatment methods: Given that music interventions alone cannot fully treat ADHD, exploring the combination of music intervention with existing therapies is an effective approach for non-invasive treatment. This includes optimizing the integration with cognitive-behavioral therapy (CBT) and pharmacological treatments to form a multimodal, synergistic intervention strategy. This integration not only helps improve the effectiveness of core symptom treatment but also enhances treatment adherence and long-term stability, addressing the limitations of single therapies and advancing music interventions from adjunctive measures to core combined strategies (Park et al., 2023).

Develop personalized music intervention platforms powered by technology. The rapid development of digital health,the development of personalized music intervention tools and platforms based on artificial intelligence, big data, and other advanced technologies is the trend. These systems could precisely adapt to individual music types, rhythm parameters, intervention duration, and frequency, thereby enhancing the targeting and applicability of interventions (Lucke-Wold et al., 2018). Smart intervention tools not only improve treatment outcomes and user engagement but also, through remote management and real-time feedback functions, greatly increase the accessibility and popularity of music interventions. This is especially important for long-term tracking and home-based interventions for children and adolescents with ADHD (Achuthan and Khobragade, 2025).
